# Underlying the Enhanced Ammonia Stress Resistance by Prebiotic Xylo-Oligosaccharides in Hybrid Groupers: An Integrative Analysis of Serum Physiology, Hepatic Transcriptomics, and Intestinal Health

**DOI:** 10.3390/ani16142178

**Published:** 2026-07-13

**Authors:** Guohua Liang, Yan Cai, Shaoqun Wang, Huizhong Shi, Ying Wu, Jianlong Li, Jingqiu Liao, Yongcan Zhou, Shifeng Wang

**Affiliations:** 1 School of Marine Biology and Fisheries, Hainan University, Haikou 570228, China; 2 Hainan Provincial Key Laboratory for Tropical Hydrobiology and Biotechnology, School of Marine Biology and Fisheries, Hainan University, Haikou 570228, China; 3 Guangxi Academy of Sciences, Nanning 530007, China

**Keywords:** prebiotic supplementation, ammonia nitrogen stress, serum enzymatic activities, hepatic functional transcriptomics, gut microbiota

## Abstract

Elevated ammonia nitrogen restricts sustainable hybrid grouper aquaculture. Though certain prebiotics relieve ammonia stress in groupers, their regulatory mechanisms remain unclear. This work investigated the protective effects and molecular mechanisms of xylo-oligosaccharides (XOSs) against ammonia stress in hybrid groupers (*Epinephelus fuscoguttatus* ♀ × *E. lanceolatus* ♂) via a 4-week feeding trial (control diet/XOS-supplemented diet) followed by a 96 h ammonia challenge. Dietary XOS significantly boosted fish growth, increasing weight gain and specific growth rate while lowering feed conversion ratio (*p* < 0.05). During ammonia exposure, XOS-fed fish exhibited markedly higher survival from 48 to 96 h, with a 40% survival gap between the two groups at 96 h. XOS elevated serum glutathione peroxidase and lysozyme activities, suppressed catalase, aspartate transaminase and alanine transaminase levels (*p* < 0.05), strengthening antioxidant capacity, non-specific immunity and hepatic health. Transcriptomics identified differentially expressed genes enriched in nitrogen metabolism, steroid biosynthesis and complement cascades, verified by qPCR. XOS mitigated ammonia-triggered intestinal injury and reshaped gut microbiota: XOS supplementation enriched beneficial genera (*Bifidobacterium*, *Turicibacter*) and suppressed pathogenic taxa (*Clostridium_sensu_stricto_1*, *Photobacterium*) under ammonia stress. Collectively, dietary XOS improves hybrid groupers growth and ammonia resistance by regulating antioxidant enzymes, immune-related genes, intestinal morphology and gut microflora. This study offers theoretical support for developing anti-ammonia feed additives for grouper farming.

## Simple Summary

Ammonia stress is a major obstacle to the sustainable development of grouper aquaculture. In this study, hybrid groupers (*E. fuscoguttatus* ♀ × *E. lanceolatus* ♂) were used as experimental fish. After a 4-week feeding trial, a 96 h ammonia stress test was conducted to explore the effects and mechanisms of xylo-oligosaccharides (XOSs) on alleviating ammonia stress in fish. The results showed that dietary XOS supplementation stabilized the survival rate of groupers under ammonia stress, optimized the antioxidant system, enhanced non-specific immunity, regulated gut microbiota composition, and reduced liver and intestinal damage. This study confirms that XOS improves the ammonia tolerance of groupers through multiple pathways, providing a theoretical basis for the development of anti-stress feed additives in aquaculture.

## 1. Introduction

*Epinephelus*, belonging to the Perciformes order, Percoidei suborder, and Epinephelinae family [1], is now known to be distributed worldwide, with 45 species found along the southeastern coast of China and in the South China Sea. The hybrid grouper is a species that is artificially crossed between two species of grouper (*E. fuscoguttatus* ♀ × *E. lanceolatus* ♂). Due to its delicious meat, rich nutrition, fast growth rate, and strong disease resistance, its market share in the grouper market has been increasing year by year.

In recent years, breakthroughs in grouper seedling breeding technology have driven the rapid expansion of farming scale, accompanied by a substantial increase in stocking density. Intensive aquaculture commonly leads to water quality deterioration, and excessive ammonia nitrogen has become a prevalent problem in aquaculture systems [2]. Numerous studies have demonstrated that accumulated ammonia nitrogen causes systemic and multi-dimensional toxic damage to fish. Physiologically, it disrupts osmotic pressure and acid base balance, induces severe oxidative stress, impairs gill respiratory structures and hepatic metabolic functions, and results in metabolic disorders and energy depletion [2]. Immunologically, it markedly suppresses the non-specific immune system, disturbs the balance of inflammatory signaling pathways, and weakens the resistance to pathogenic bacteria [3,4]. Furthermore, ammonia stress dramatically alters the intestinal microbial community, reduces the abundance of beneficial microbiota while enriching opportunistic pathogens [5], and destroys the integrity of the intestinal barrier. These changes further exacerbate oxidative damage and immunosuppression, ultimately leading to growth retardation, reduced disease resistance and increased mortality. Accordingly, it is urgent to develop strategies to improve the stress tolerance of fish, and functional feed additives serve as an effective solution.

Prebiotics, non-digestible substances that selectively stimulate beneficial gut microbes to enhance host immunity, stress resistance, and health, are promising alternatives to antibiotics [6]. Xylo-oligosaccharides (XOSs), emerging prebiotics composed of β-(1→4)-linked xylose chains [7], exhibit multiple health benefits in mammals, including promoting beneficial gut bacteria proliferation, increasing short-chain fatty acid production, and exerting immunomodulatory, antioxidant, anti-inflammatory, and antibacterial effects [8,9,10]. In aquatic animals, XOS improves growth performance and digestive enzyme activity in gibel crucian carp [11], enhancing serum lysozyme activity, complement activity, phagocytosis, and pathogen resistance [12,13,14]. However, most current investigations are limited to basic phenotypic observations. The mechanisms underlying the protective effects of XOS against ammonia stress in fish remain unclear. There is a lack of integrated verification from multiple perspectives.

In this study, hybrid groupers were fed XOS-supplemented diets for 4 weeks to explore the influences of XOS on physiological indices during a 96 h ammonia nitrogen stress challenge. Samples collected before and after 24 h ammonia exposure were used for transcriptomic and microbial diversity analysis. We systematically investigated how XOS modulates the antioxidant system, non-specific immunity, hepatic gene expression and gut microbiota in stressed fish so as to explore the multi-dimensional mechanisms of XOS in alleviating ammonia toxicity. The findings provide a theoretical basis for XOS application as a feed additive to mitigate ammonia nitrogen damage and reduce economic losses in hybrid grouper aquaculture.

## 2. Materials and Methods

### 2.1. Feed Preparation and Storage

XOSs were purchased from Shandong Shengyuan Biotechnology Co., Ltd. (Jinan, China). Two types of diets were prepared: a control diet (Group C) and an experimental diet containing 0.05% xylo-oligosaccharide (Group X). For Group X, 0.5 g of xylo-oligosaccharide was dissolved in 50 mL of sterile water, and the solution was sprayed onto 1 kg of commercial feed (Guangdong Evergreen Aquatic Products Co., Ltd. (Zhanjiang, China), the specific components of the commercial feed are shown in Table 1) using a spray bottle with continuous stirring for uniform distribution. Group C was treated identically but with sterile water only. The treated feeds were air-dried at room temperature before storage at 4 °C, and fresh batches of both diets were prepared weekly to maintain quality.

### 2.2. Fish Rearing and Management

Healthy juvenile hybrid groupers (*E. fuscoguttatus* ♀ × *E. lanceolatus* ♂) were purchased from Haitou grouper breeding base in Hainan Province, with an average weight of 84.10 ± 1.98 g. The feeding experiment was carried out in the culture room of Hainan Danzhou Yuhai Lanke Biotechnology Co., Ltd. (Danzhou, China). The hybrid groupers were acclimated for a week prior to the formal experiments. During the acclimation period, fish were fed basal feed (Guangdong Evergreen Aquatic Products Co., Ltd.). Water condition was maintained as follows: temperature between 28 and 32 °C, pH between 7.6 and 8.2, dissolved oxygen at or above 6.5 mg/L, and salinity between 27.5‰ and 32‰.

Healthy fish were randomly allocated into a control group (Group C) and an experimental group (Group X), each consisting of six replicates (40 fish/replicate). In each group, three replicates were assigned for survival monitoring (no samples were taken through the feeding trial and the ammonia nitrogen stress test) and the remaining three for sampling. Feeding is given daily at 9:00 a.m. and 5:00 p.m., at a rate of 2% of the fish’s body weight per day. This was continued for 4 weeks.

All fish were weighed at the beginning and end of the feeding trial (Day 0 and Day 28). The weight gain rate (WGR), specific growth rate (SGR), and feed conversion ratio (FC) were calculated using the following formulae:

WGR (%) = [(W_final − W_initial)/W_initial] × 100

SGR (%/d) = (ln W_final − ln W_initial)/d × 100

FC = FI/(W_final − W_initial)

where W_final and W_initial represent the final and initial mean body weights, respectively, and d is the duration of the feeding trial (days).

### 2.3. Ammonia Nitrogen Stress Test

After the 4-week feeding trial, all hybrid groupers were fasted for 24 h prior to the stress challenge. Based on the results of our previous published study [15], the median lethal concentration (LC_50_) of total ammonia nitrogen (TAN) was determined to be 1.4 mg/L (pH = 6.8 ± 0.2; water temperature = 24 ± 1 °C). Therefore, this concentration was adopted for the formal experiment. Ammonium chloride (Xilong Science Co., Ltd., Guangzhou, China) was used to prepare the test water. During the 96 h ammonia nitrogen stress period, both groups were subjected to identical environmental conditions: water temperature, pH and ammonia concentration were measured every 2 h, and ammonium chloride (NH_4_Cl) solution was supplemented to maintain a stable level of non-ionic ammonia for both the control and XOS-fed groups. Fish survival was recorded every 12 h.

The cumulative survival rate (CSR) was calculated as follows.

CSR (%) = 100 × number of survival fish after the stress test/number of fish before the stress test.

### 2.4. Sample Collection and Processing

**Figure 1:**
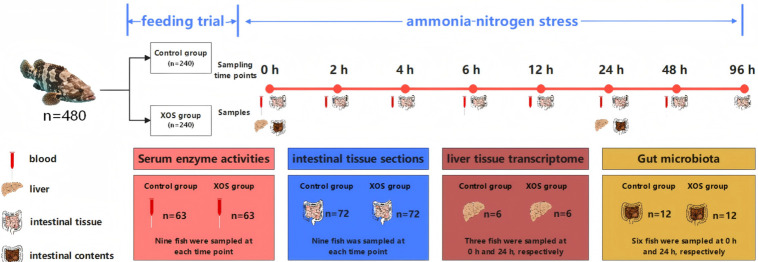
Experimental design and sampling method.

The sample collection and processing in this experiment are shown in Figure 1. At each sampling time point (0, 2, 4, 6, 12, 24, 48 and 96 h after ammonia nitrogen stress test started), nine fish were randomly selected from sampling replicates of Group C and Group X, respectively, for simultaneous multi-tissue sampling. Blood was collected from the caudal vein using sterile syringes at 0, 2, 4, 6, 12, 24 and 48 h only. After clotting at room temperature for 2 h, the samples were centrifuged at 3000× *g* for 15 min at 4 °C to separate serum, which was stored at −80 °C for the determination of enzyme activities. Meanwhile, approximately 1 cm of midgut tissue was dissected from the same fish at each designated time point and immediately fixed in 4% paraformaldehyde at 4 °C for 24 h. Thereafter, the fixed tissues were processed for paraffin embedding, sectioning, HE staining, and microscopic imaging by Servare Biotechnology Co., Ltd. (Foshan, China).

Furthermore, at the key time points of 0 h and 24 h, liver tissues were collected from three individuals, and intestinal contents were aseptically harvested from another six individuals among the above-mentioned nine sampled fish per group. The collected liver tissues were immediately flash-frozen in liquid nitrogen and stored at −80 °C for subsequent RNA extraction and transcriptome sequencing. The intestinal content samples were snap-frozen in dry ice and preserved at −80 °C for subsequent DNA extraction and gut microbiota analysis.

No additional fish were sampled, and all experimental samples were derived from the same batch of fish at each time point to avoid individual differences and redundant animal consumption.

### 2.5. DNA Extraction, Illumina Sequencing, and Microbiome Analysis

The intestinal content samples obtained above were sent to Shanghai Majorbio Biomedical Technology Co., Ltd. (Shanghai, China). for microbial diversity analysis using its Illumina Nextseq 2000 high-throughput sequencing platform. Purified amplicons were pooled in equimolar amounts and subjected to paired-end sequencing on the Illumina Nextseq 2000 platform in accordance with the manufacturer’s protocols. Experimental procedures, including DNA extraction, sequencing and data analysis, were performed following the methods described in previous research [16]. Data analysis included α-diversity, β-diversity and community composition analyses, all of which were conducted according to the aforementioned methods.

### 2.6. Determination of Physiological Indicators

The serum samples (0 h, 2 h, 4 h, 6 h, 12 h, 24 h,48 h), which had been stored in a refrigerator at −80 °C, were thawed on ice without any intervention. Following the addition of reagents in accordance with the kit instructions, the absorbance values were measured and recorded using an enzyme marker (manufacturer: Thermo, Waltham, MA, USA, model: 5580). Thereafter, the enzyme activity indexes were calculated in accordance with the instructions. The following indices were subsequently measured: malondialdehyde (MDA), glutathione peroxidase (GSH-Px), superoxide dismutase (SOD), catalase (CAT), total antioxidant capacity (T-AOC), cortisol (COR), glucose (Glc), lactate (LD), lysozyme (LZM), alkaline phosphatase (AKP), total protein (TP), albumin (ALB), aspartate transaminase (AST), and alanine transaminase (ALT).

The enzyme activity assay kits utilized in this experiment were procured from Nanjing Jiancheng Bioengineering Institute Co., Ltd (Nanjing, China).

### 2.7. RNA Extraction, RNA Sequencing, and Transcriptomic Analysis

Liver tissue samples collected from the ammonia nitrogen stress 0 h control group, ammonia nitrogen stress 0 h Xylo-oligosaccharide group, ammonia nitrogen stress 24 h control group and ammonia nitrogen stress 24 h Xylo-oligosaccharide group were sent to Shanghai Majorbio Bio-Pharm Technology Co., Ltd. (Shanghai, China) for high-throughput transcriptome sequencing.

Total RNA was extracted from the liver tissue samples (reagent: Eastep Super Total RNA Extraction Kit (Commodity No.: LS1040), Shanghai Plomag Bio-Products Co., Ltd. (Shanghai, China)), the concentration and purity of the extracted RNA were determined using an Ultra-Micro Nucleic Acid and Protein Assay Instrument (Model: Nano-800+, manufacturer: Shanghai Jiapeng Science and Technology Co., Ltd. (Shanghai, China)), and the integrity of the RNA was detected by agarose gel electrophoresis. The Agilent 5300 fragment analyzer system (model: 5300, manufacturer: Agilent Technologies, Inc. Santa Clara, CA, USA) was used to determine the RQN values and A–T base pairing with ployA using magnetic beads with oligo (dT) was used to isolate mRNA from total RNA for transcriptome analysis. Enrichment of the mRNA was obtained and the selection of suitable conditions and random fragmentation of the mRNA into small fragments of approximately 300 bp was completed. Synthesis of single-stranded cDNA by reverse transcription using mRNA as a template was completed, followed by two-strand synthesis to form a stable double-stranded structure; the double-stranded cDNA structure has a sticky end patched to a flat end with an A base at the 3′ end followed by an adapter sequence. The ligated adapter product was purified and fragment sorted, and the sorted product was used for PCR amplification and purification to obtain the final library; the NovaSeq X Plus platform (Illumina, Inc., San Diego, CA, USA). was used for online sequencing.

Software Fastp (0.20.0) was used for raw data quality control and quality control data analysis. After obtaining high-quality sequencing data, all clean data were de novo assembled using Trinity version 2.4.0 and the assembly results were evaluated to obtain reference sequences for subsequent analysis. All genes and transcripts obtained from transcriptome assembly were compared with six databases (NR, Swiss-prot, Pfam, EggNOG, GO and KEGG databases) to obtain comprehensive functional information and statistics on annotation of each database. RSEM, an expression quantification software, was used to quantify the expression levels of the genes and transcripts. DESeq2 (version 1.10.1) was used to perform the analysis of the differential expression of genes/transcripts between samples or groups to identify the differentially expressed genes/transcripts. GO enrichment and KEGG enrichment analyses were performed on the genes in the gene set using the software Goatools version 1.2.3.

### 2.8. qPCR Verification

Eight genes with significant differences were randomly selected for qPCR verification of the transcriptome, with β-actin used as the internal reference gene. The primers were designed using the software DNAMAN 9.0 and Primer 5.0, and the synthesis was carried out by Beijing Tsingke Biotech Co., Ltd (Beijing, China). The qPCR instrument (model: Q2000B) was manufactured by LongGene Scientific Instrument Co., Ltd. (Hangzhou, China), a company based in Hangzhou, China. The primer sequences are presented in Table 2 for reference. Three replicates were conducted for each gene, and the relative expression level was calculated using the 2^−ΔΔCt^ relative quantification method.

### 2.9. Data Processing and Analysis

Test data were compiled in Microsoft Office Excel 2007. One-way analysis of variance (ANOVA) combined with Duncan’s multiple range test was performed using SPSS 20.0 (SPSS Inc., Chicago, IL, USA). For enzyme activity data, two separate sets of one-way ANOVA were conducted: comparisons among different treatment groups at the same sampling time point, and comparisons across different time points within each individual treatment group. Conventional statistical methods were applied for the rest of the measured indicators. Differences with *p* < 0.05 were regarded as statistically significant. All data are presented as mean ± standard deviation (mean ± SD).

## 3. Results

### 3.1. Effects of Dietary Xylo-Oligosaccharides (XOSs) on Growth Performance and Ammonia Nitrogen Stress Tolerance of Hybrid Groupers

**Figure 2:**
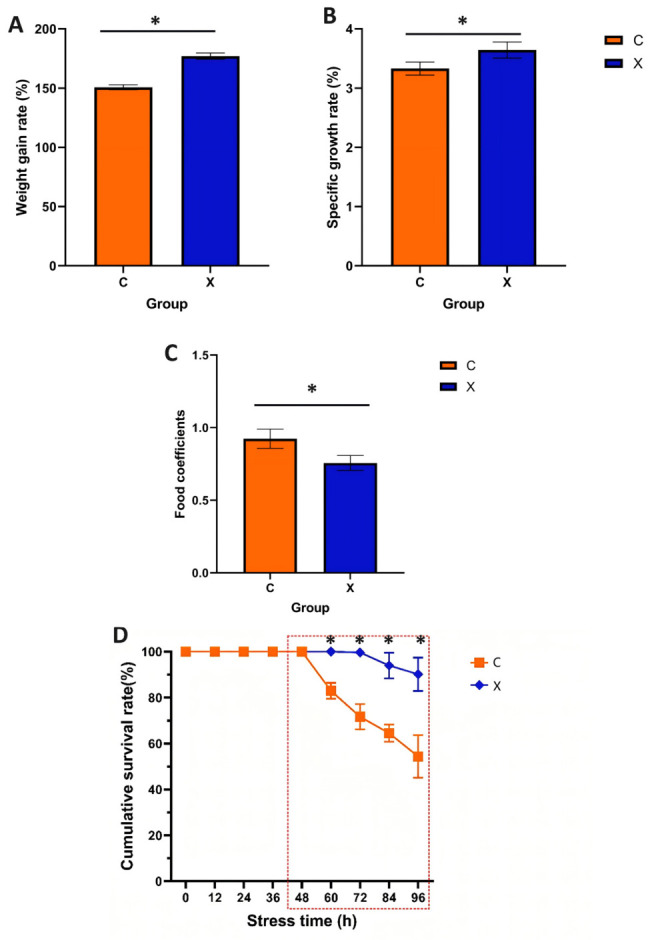
Effects of dietary xylo-oligosaccharide (XOS) supplementation on growth performance and ammonia nitrogen stress tolerance in fish. (**A**) Weight gain rate (%); (**B**) Specific growth rate (%). (**C**) Feed conversion ratio. (**D**) Cumulative survival rate (%) during ammonia nitrogen stress, recorded at 12 h intervals, and the red dashed box marks the time points of divergent mortalities between groups. All data are expressed as mean ± standard deviation (SD). * *p* < 0.05. C, control group (basal diet); X, XOS-supplemented group (basal diet + XOS).

Dietary XOS supplementation significantly enhanced the growth performance of hybrid groupers. As presented in Figure 2A,B, Group X showed a significantly higher weight gain rate and specific growth rate than Group C (*p* < 0.05). Moreover, the feed conversion ratio of Group X was substantially lower than that of Group C (Figure 2C), suggesting that XOS supplementation improved feed utilization efficiency.

As shown in Figure 2D, the effect of 96 h ammonia nitrogen stress on the cumulative survival rate of hybrid groupers was investigated in this study. During the first 36 h of ammonia nitrogen stress, the survival rates of both the control and XOS-fed groups remained high and comparable (*p* > 0.05). However, from 48 h onward, a marked divergence emerged between the two groups. At 60 h, the survival rate of Group C dropped sharply to 82.5%, while that of the XOS group remained at 100.0% (*p* < 0.05). This gap continued to widen over time: at 72 h, survival rates were 72.5% in Group C versus 99.0% in Group X (*p* < 0.05), and, at 96 h, Group C exhibited only 52.5% survival, whereas Group X maintained a survival rate of 92.5% (*p* < 0.05). These results demonstrate that dietary XOS supplementation significantly improves the survival of hybrid groupers under high ammonia nitrogen stress, with the protective effect becoming increasingly pronounced over time.

### 3.2. Effects of Dietary Xylo-Oligosaccharides on the Physiology of Hybrid Groupers Under Ammonia Nitrogen Stress

**Figure 3:**
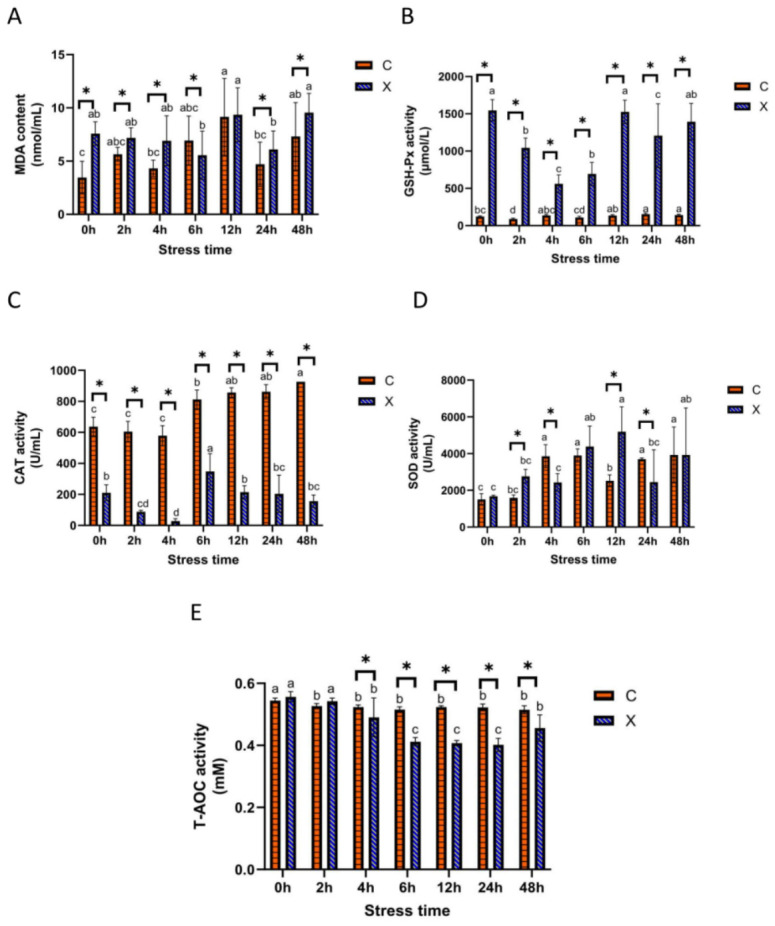
Effects of ammonia nitrogen stress on the antioxidant capacity of hybrid groupers. (**A**) MDA, (**B**) GSH-PX, (**C**) CAT, (**D**) SOD, and (**E**) T-AOC. Lowercase letters are used to compare differences between different time points in the same treatment, and * is used to compare differences between different treatments at the same time. Values are expressed as mean ± standard deviation (SD), and a different letter above the bar indicates a significant difference (*p* < 0.05). C, control group (fish fed a basal diet); X, XOS-supplemented group (fish fed a basal diet supplemented with xylo-oligosaccharides).

Following a four-week period of feeding hybrid groupers with xylo-oligosaccharides, the antioxidant indicators of hybrid groupers in the experimental and control groups are presented in Figure 3. As illustrated in Figure 3A, the MDA content in Group C exhibited a gradual increase following ammonia nitrogen stress, reaching its peak at 12 h and subsequently declining and stabilizing. In contrast, the MDA content in Group X demonstrated a gradual decrease following ammonia nitrogen stress, reaching its lowest point at 6 h and subsequently increasing significantly (*p* < 0.05). Figure 3B illustrates that the GSH-Px activity in Group X was markedly higher than that in the control group at all time points (*p* < 0.05). Additionally, the GSH-Px activity in Group X exhibited a decline at 2 and 4 h following ammonia nitrogen stress, reaching its lowest point at 4 h, subsequently demonstrating a notable increase and stabilization (*p* < 0.05). As illustrated in Figure 3C, the CAT activity in Group X was markedly diminished in comparison to the control group at all time points (*p* < 0.05). The CAT activity reached its lowest level at the four-hour mark of ammonia nitrogen stress, subsequently demonstrating a significant increase after four hours, reaching its peak at the six-hour mark, and then exhibiting a gradual decline. In Group C, the CAT activity exhibited a notable increase (*p* < 0.05) after four hours of ammonia nitrogen stress, subsequently stabilizing. As illustrated in Figure 3D, the SOD activity in Group C exhibited a gradual increase from 0 h to 6 h of ammonia nitrogen stress, reaching a significant peak at 6 h (*p* < 0.05). Thereafter, it demonstrated a gradual decrease, reaching the lowest point at 12 h (*p* < 0.05), before showing a significant increase (*p* < 0.05) and stabilizing. In Group X, the SOD activity began to gradually increase at 0 h of ammonia nitrogen stress, reaching a significant peak at 12 h (*p* < 0.05), before showing a gradual decrease. As illustrated in Figure 3E, the T-AOC activity in Group X exhibited a notable decline from the second hour following ammonia nitrogen stress, reaching a nadir at the 24th hour. Thereafter, a marked ascendance was observed. The T-AOC activity at 6, 12 and 24 h was found to be statistically significant.

**Figure 4:**
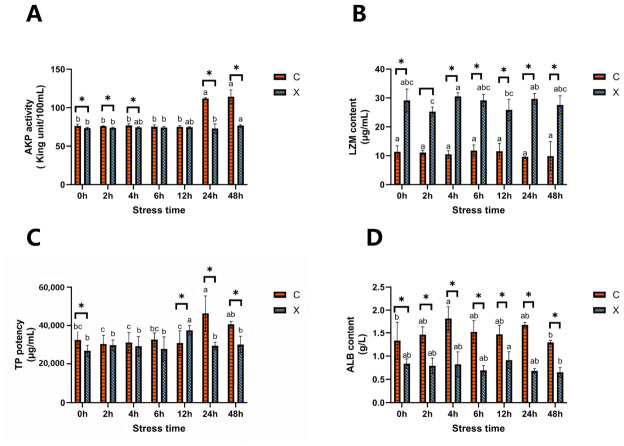

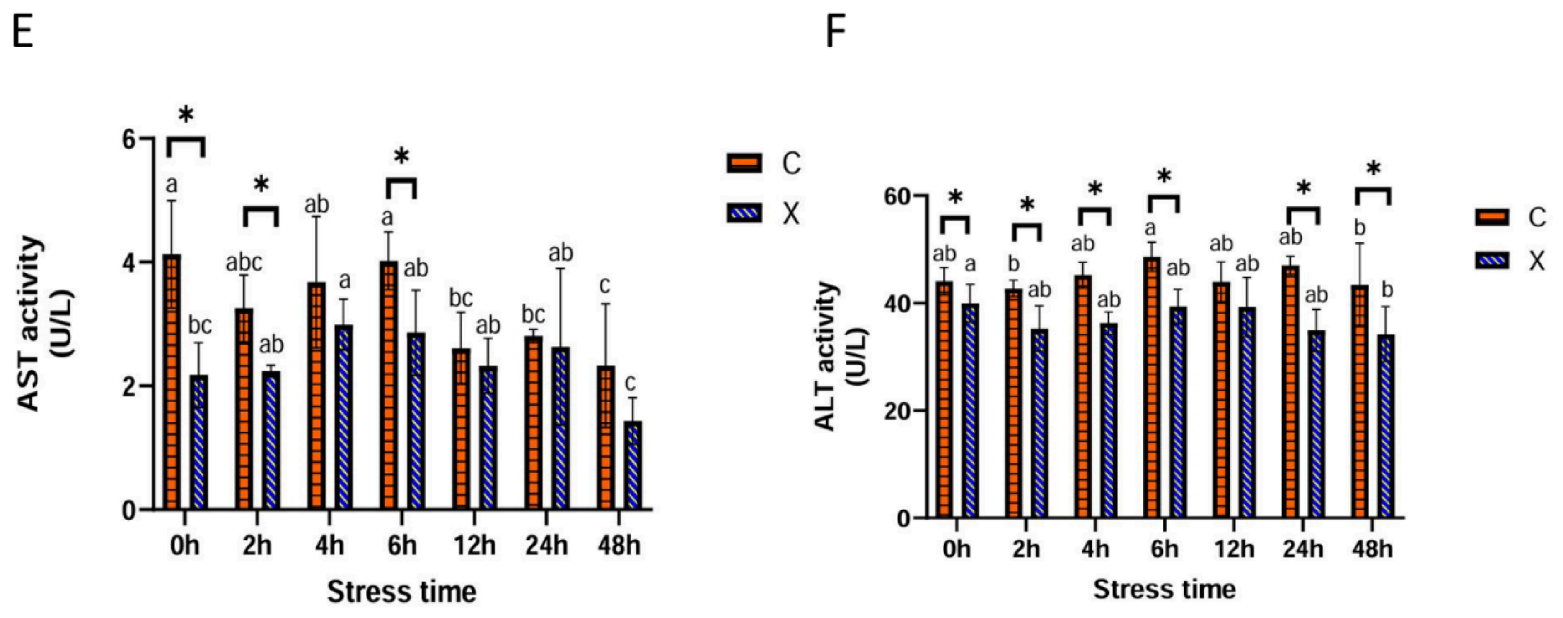
Effects of ammonia nitrogen stress on non-specific immunity of hybrid groupers. (**A**) AKP, (**B**) LZM, (**C**) total protein TP, (**D**) albumin ALB, (**E**) AST, and (**F**) ALT. Lowercase letters are used to compare differences between different time points in the same treatment, and * is used to compare differences between different treatments at the same time. Values are expressed as mean ± standard deviation (SD), and a different letter above the bar indicates a significant difference (*p* < 0.05). C, control group (fish fed a basal diet); X, XOS-supplemented group (fish fed a basal diet supplemented with xylo-oligosaccharides).

Following a four-week period during which the fish were fed XOS, the results of the non-specific immune indexes for the experimental and control groups are presented in Figure 4. As illustrated in Figure 4A, the AKP activity of Group X demonstrated an increase in ammonia nitrogen stress at 48 h, while the AKP activity of Group C exhibited a significant rise at both 24 and 48 h following ammonia nitrogen stress, resulting in a notable elevation compared to that of Group X (*p* < 0.05). Figure 4B illustrates that the LZM activity of Group X was significantly higher than that of Group C at each time point (*p* < 0.05). Following ammonia nitrogen stress, there was a decrease in LZM activity, followed by an increase and subsequent stability during the 0–4 h period. In contrast, the LZM activity of Group C exhibited no significant change during the 0–48 h stress period. As illustrated in Figure 4C, the TP content of Group X exhibited a gradual increase from 2 h of ammonia nitrogen stress, reaching a peak at 12 h and subsequently declining before stabilizing. In contrast, the TP content of Group C reached its peak at 24 h of ammonia nitrogen stress, demonstrating a significantly higher concentration than that of Group X (*p* < 0.05). As illustrated in Figure 4D, the ALB content in Group X was markedly higher than that in Group C at all time points (*p* < 0.05). Figure 4E illustrates that the AST activity of Group X was consistently lower than that of Group C and significantly lower than that of Group C from 0 h to 6 h after ammonia nitrogen stress (*p* < 0.05). Additionally, the change trend of AST activity of Group X at each time point was found to be largely aligned with that of Group C. As illustrated in Figure 4F, the ALT activity of Group X was markedly lower than that of Group C (*p* < 0.05). Moreover, the change trend of ALT activity in Group X at each time point was largely consistent with that of Group C.

**Figure 5:**
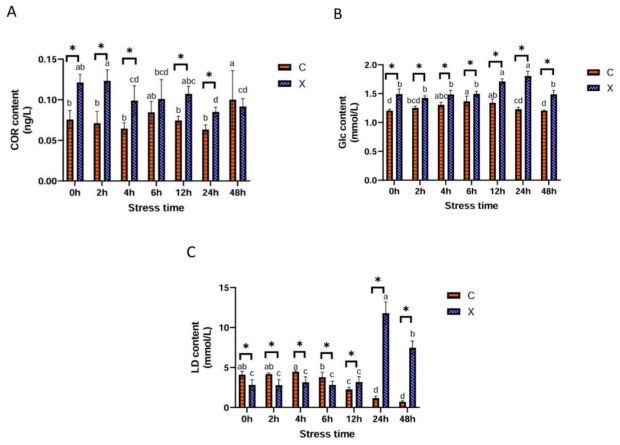
Effects of ammonia nitrogen stress on the stress ability of hybrid groupers. (**A**) Cortisol (COR), (**B**) glucose (Glc), and (**C**) lactate dehydrogenase (LD). Lowercase letters are used to compare differences between different time points in the same treatment, and * is used to compare differences between different treatments at the same time. Values are expressed as mean ± standard deviation (SD), and a different letter above the bar indicates a significant difference (*p* < 0.05). C, control group (fish fed a basal diet); X, XOS-supplemented group (fish fed a basal diet supplemented with xylo-oligosaccharides).

Following the administration of xylo-oligosaccharides to hybrid groupers over a four-week period, the stress index results for the experimental and control groups are presented in Figure 5. Figure 5A illustrates the COR content. The COR content of Group X was found to be significantly higher than that of Group C at all time points from 0 h to 24 h following ammonia nitrogen stress (*p* < 0.05). The Glc content is illustrated in Figure 5B. Following the imposition of ammonia nitrogen stress, the Glc content of Group C exhibited a pattern of initial increase, followed by a decline. It subsequently demonstrated a significant surge, reaching its peak at the 6 h mark (*p* < 0.05). The LD content is illustrated in Figure 5C. Following ammonia nitrogen stress, the LD content in Group C exhibited a declining trend, whereas the LD content in Group X demonstrated a notable surge, reaching its peak at 24 h (*p* < 0.05). During the initial 6 h period after ammonia nitrogen stress, the LD content in the Group X was markedly lower than that of Group C. However, between 12 and 48 h after ammonia nitrogen stress, the LD content in Group X surpassed that of the control group (*p* < 0.05).

### 3.3. Transcriptome Changes Were Induced by Xylo-Oligosaccharides and Ammonia Stress

#### 3.3.1. GO Functional Annotation and Enrichment Analysis of Differentially Expressed Genes

**Figure 6:**
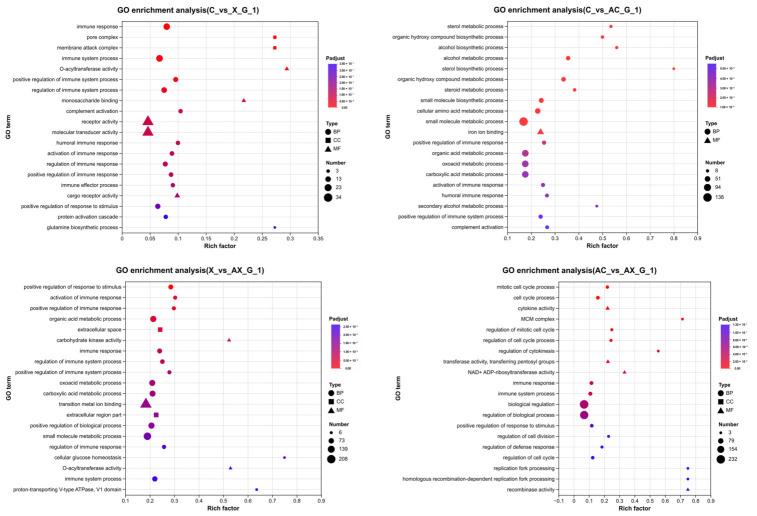
GO functional annotation enrichment analysis. The vertical axis represents GO term and the horizontal axis represents rich factor. The larger the rich factor, the greater the degree of enrichment; the size of the bubble indicates the number of genes in this GO term, the shape of the bubble represents different types, and the color of the bubble corresponds to different FDR (Pvaule _corrected) ranges. BP: biological process, CC: cellular component, MF: molecular function. C, control group (fish fed a basal diet); X, XOS-supplemented group (fish fed a basal diet containing xylo-oligosaccharides); AC, control group exposed to ammonia nitrogen stress; AX, XOS-supplemented group exposed to ammonia nitrogen stress.

Through GO function enrichment analysis of the differential genes in the four groups (Figure 6), the top 20 enriched GO terms in the C vs. X comparison included immune response, immune system process, positive regulation of immune system process, monosaccharide binding, receptor activity and molecular transducer activity. The top 20 enriched GO terms in the C vs. AC comparison included alcohol metabolic process, organic hydroxy compound metabolic process, cellular amino acid metabolic process, small molecule metabolic process and iron ion binding. The top 20 enriched GO terms in the X vs. AX comparison included positive regulation of response to stimulus, organic acid metabolic process, extracellular space, immune response, regulation of immune system process, oxoacid metabolic process and transition metal ion binding. The top 20 enriched GO terms in the AC vs. AX comparison included mitotic cell cycle process, cell cycle process, immune response, immune system process, biological regulation and regulation of biological process.

The above GO enrichment results indicate that dietary xylo-oligosaccharides (XOSs) enhance the immune response of fish under normal conditions. Ammonia nitrogen stress mainly disrupts basal metabolism and ion homeostasis. Under ammonia nitrogen exposure, XOS regulates cell cycle and immune response, thereby effectively alleviating ammonia-induced damage.

#### 3.3.2. KEGG Functional Annotation and Enrichment Analysis of Differentially Expressed Genes

**Figure 7:**
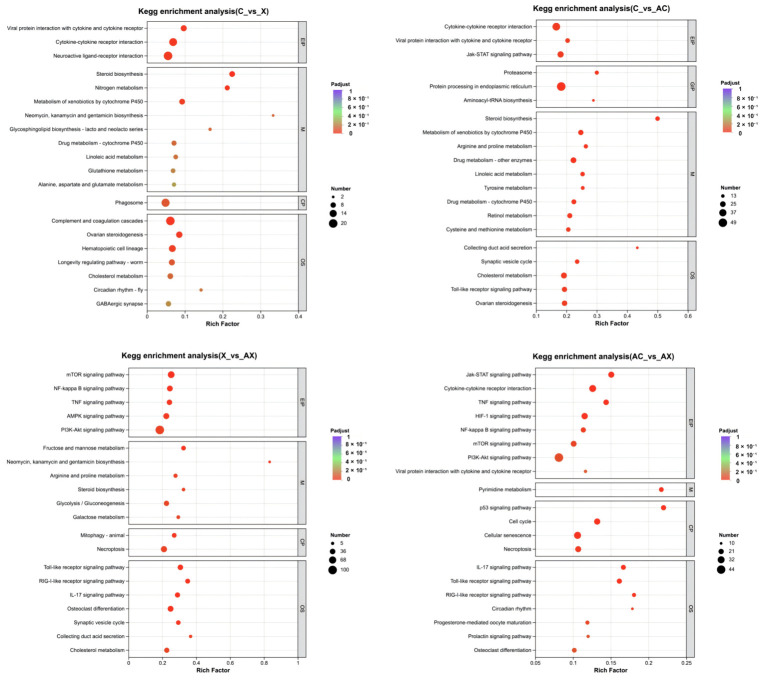
KEGG pathway enrichment map. The abscissa is the enrichment rate, and the default parameter of the ordinate is Padjust. Each bubble in the figure represents a KEGG pathway. The size of the bubble is proportional to the number of unigenes enriched by the KEGG pathway, and the position of the bubble represents different pathway types: metabolism (M), genetic information processing (GIP), environmental information processing (EIP), cellular process (CP), organism system (OS) and drug development (DD). C, control group (fish fed a basal diet); X, XOS-supplemented group (fish fed a basal diet containing xylo-oligosaccharides); AC, control group exposed to ammonia nitrogen stress; AX, XOS-supplemented group exposed to ammonia nitrogen stress.

The KEGG metabolic pathway enrichment analysis of the four groups is shown in Figure 7. The top 20 enriched metabolic pathways in the C vs. X comparison included steroid biosynthesis, nitrogen metabolism, complement and coagulation cascades, viral protein interaction with cytokine and cytokine receptor, and cytokine–cytokine receptor interaction. The results showed that, under normal conditions, XOS modulated steroid biosynthesis, nitrogen metabolism, and immune pathways related to complement and cytokines.

The top 20 enriched metabolic pathways in the C vs. AC comparison included steroid biosynthesis, collecting duct acid secretion, proteasome, and metabolism of xenobiotics by cytochrome P450. This indicated that ammonia nitrogen stress significantly affected steroid biosynthesis, substance transport, xenobiotic detoxification and protein degradation.

The top 20 enriched metabolic pathways in the X vs. AX comparison included Toll-like receptor signaling pathway, mTOR signaling pathway, and RIG-I-like receptor signaling pathway. Under ammonia nitrogen stress, XOS supplementation mainly activated innate immune receptor pathways including TLR and RIG-I-like receptor pathways, as well as the mTOR pathway associated with nutrient sensing and stress regulation.

The top 20 enriched metabolic pathways in the AC vs. AX comparison included the p53 signaling pathway and Jak-STAT signaling pathway. This demonstrates that, under ammonia nitrogen stress, XOS modulates the p53 pathway involved in cell apoptosis and cell cycle, as well as the canonical Jak-STAT immune signaling pathway, ultimately conferring protective effects on fish.

#### 3.3.3. Results of qPCR Verification

**Figure 8:**
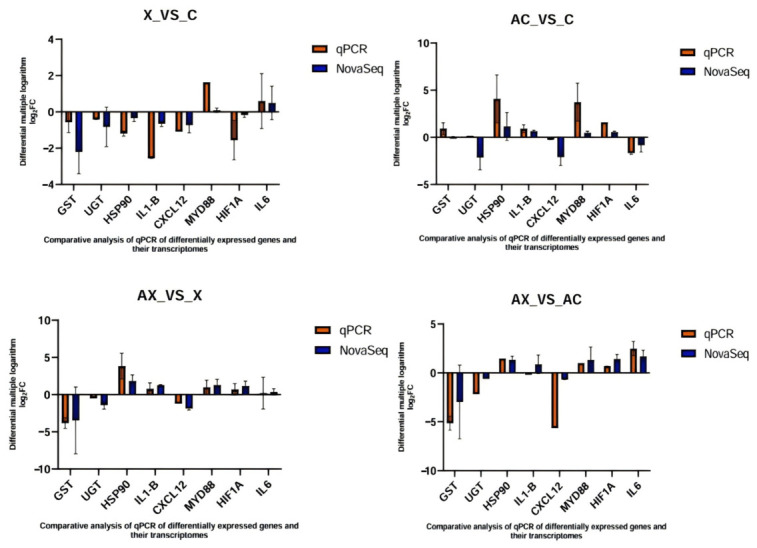
Comparative analysis of qPCR and transcriptome for four groups. Values are expressed as mean ± standard deviation (SD). C, control group (fish fed a basal diet); X, XOS-supplemented group (fish fed a basal diet containing xylo-oligosaccharides); AC, control group exposed to ammonia nitrogen stress; AX, XOS-supplemented group exposed to ammonia nitrogen stress.

In order to verify the transcriptome, eight differentially expressed genes were randomly selected for qPCR, and the results are shown in the following figure (Figure 8): the up-regulation or down-regulation trend shown by qPCR is consistent with the transcriptome results, indicating that the transcriptomic analysis is authentic and credible.

### 3.4. Effects of Ammonia Nitrogen Stress and Xylo-Oligosaccharides on Intestinal Health of Hybrid Groupers

**Figure 9:**
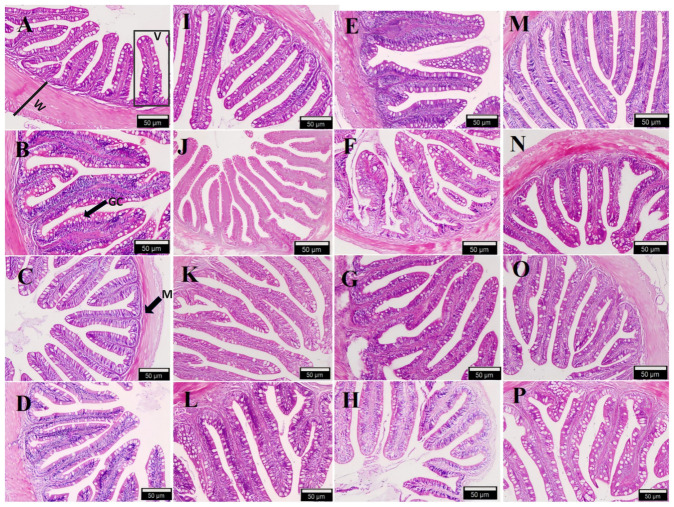
Effects of ammonia stress and XOS on intestinal slices of grouper (200×). (**A**) Group C 0 h, (**B**) Group C 2 h, (**C**) Group C 4 h, (**D**) Group C 6 h, (**I**) Group X 0 h, (**J**) Group X 2 h, (**K**) Group X 4 h, (**L**) Group X 6 h, (**E**) Group C 12 h, (**F**) Group C 24 h, (**G**) Group C 48 h, (**H**) Group C 96 h, (**M**) Group X 12 h, (**N**) Group X 24 h, (**O**) Group X 48 h, and (**P**) Group X 96 h. W: intestinal wall (as shown by the black bar); M: intestinal mucosa (as indicaded by the black arrow); V: intestinal villi (as indiated by the black box); GC: goblet cells (as indicaded by the black arrow). C, control group (fish fed a basal diet); X, XOS-supplemented group (fish fed a basal diet supplemented with xylo-oligosaccharides).

The histopathological results of the intestine as affected by ammonia nitrogen stress are shown in Figure 9. At 0 h, 2 h and 4 h of ammonia nitrogen stress, the intestinal tissues of grouper in Groups C and X were more structurally intact, with good mucosal integrity, thicker intestinal walls, intestinal villi intact, and goblet cells orderly distributed on the intestinal villi. When ammonia nitrogen stress was applied for 6 h, Group C showed a slight apoptosis of mucosal cells in the intestinal wall, an increase in inflammatory cells in the intestinal wall, a slight breakage of intestinal villi, and a disordered arrangement of goblet cells, while Group X showed no obvious changes. When the ammonia nitrogen stress was 12 h, some of the intestinal villi in Group C started to break and fall off, the number of hemocytes in the intestinal wall increased significantly, and apoptosis appeared in the intestinal wall mucosal cells; there was no obvious change in Group X. When ammonia and nitrogen stress was at 24 h, the intestinal wall of Group C became thin, a large number of intestinal villi were broken off, and a large number of mucosal cells were apoptotic; in Group X, a slight apoptosis of intestinal mucosal cells was observed, the intestinal villi appeared to be slightly broken, and there were a large number of hematocrits aggregated in the intestinal wall. When ammonia nitrogen stress was at 48 h, the integrity of the intestinal wall of Group C was seriously damaged, a large number of inflammatory cells were gathered, the thickness of the intestinal wall was sharply reduced, a large number of intestinal villi were detached, and a large number of inflammatory cells were gathered, while the thickness of the intestinal wall of Group X was reduced, the number of goblet cells on the intestinal villi was reduced, and the intestinal wall was slightly broken. At 96 h of ammonia nitrogen stress, the thickness of the intestinal wall of Group C was sharply reduced, the number of goblet cells on the intestinal villi was reduced in large numbers, and a large number of mucosal cells were apoptotic, while the intestinal villi of Group X were broken and began to break and fall off, and slight apoptosis of mucosal cells occurred.

**Figure 10:**
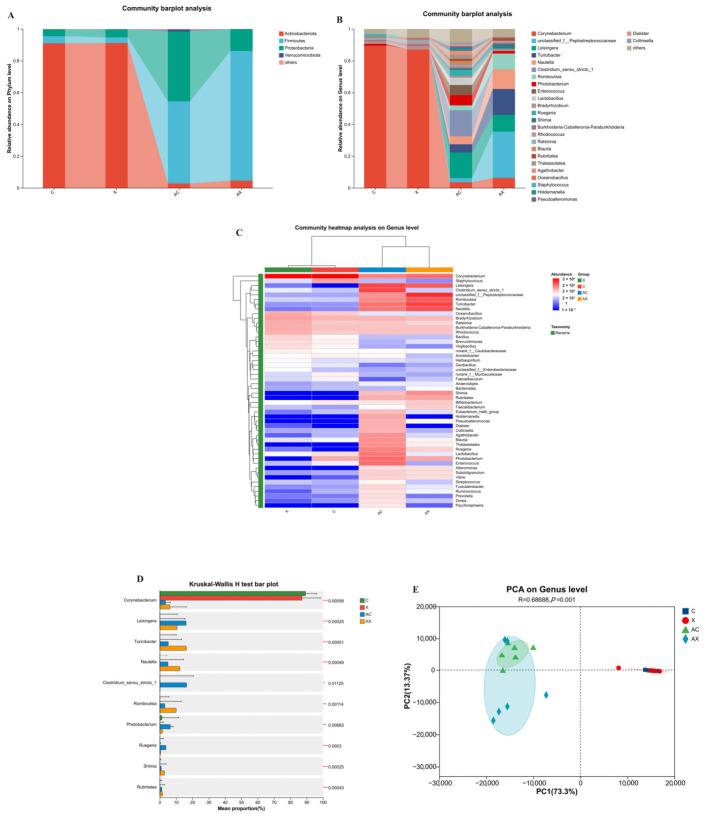
Effects of ammonia nitrogen stress and XOS on the gut microbiota of grouper. (**A**) Relative abundance of gut microbiota in grouper at the phylum level. (**B**) Relative abundance of gut microbiota in grouper at the genus level. (**C**) Heat map of gut microbial community abundance in grouper. (**D**) Multi-species test of variance bar chart, * represents *p* < 0.05, ** represents *p* < 0.01, *** represents *p* < 0.001. (**E**) PCA analysis of intestinal samples of grouper. The 95% confidence ellipses clearly separated samples from distinct groups, indicating obvious transcriptional differences between treatments. C, control group (fish fed a basal diet); X, XOS-supplemented group (fish fed a basal diet containing xylo-oligosaccharides); AC, control group exposed to ammonia nitrogen stress; AX, XOS-supplemented group exposed to ammonia nitrogen stress.

As shown in Figure 10A, the phyla with the highest relative abundance in Group C were Actinobacteriota (90.34%), Firmicutes (4.39%), and Proteobacteria (4.77%); the dominant phyla in Group X were Actinobacteriota (88.86%), Firmicutes (4.29%), and Proteobacteria (6.23%); the dominant phyla in Group AC were Actinobacteriota (6.13%), Firmicutes (50.76%), and Proteobacteria (41.14%); and the phyla with the highest relative abundance in Group AX were Actinobacteriota (7.72%), Firmicutes (57.96%), and Proteobacteria (31.98%). As shown in Figure 10B, the dominant genera in Group C were *Corynebacterium* (89.31%), *Staphylococcus* (1.71%), *Photobacterium* (1.29%), and *Bradyrhizobium* (1.05%); the dominant genera in Group X were *Corynebacterium* (87.13%), *Bradyrhizobium* (1.74%), *Oceanobacillus* (1.37%), *Ralstonia* (1.31%), and *Burkholderia-Caballeronia-Paraburkholderia* (1.3%); the dominant genera in Group AC were *Clostridium_sensu_stricto* (16.39%), *Leisingera* (16.12%), *Photobacterium* (6.45%), *Enterococcus* (6.38%), and *Turicibacter* (5.17%); and the dominant genera in Group AX were *unclassified_f_Peptostreptococcaceae* (29.19%), *Turicibacter* (16.24%), *Nautella* (12.29%), *Leisingera* (10.54%), and *Romboutsia* (10%). As shown in Figure 10C, *Corynebacterium* was the most abundant genus in both Group C and Group X, while *Psychrosphaera*, *Alteromonas*, *Thalassotalea*, *Pseudoalteromonas*, *Holdemanella*, *Rubritalea* and *Shimia* showed the lowest abundance. Compared with Group C, Group X exhibited significantly increased abundance of *Oceanobacillus*, *Bradyrhizobium*, *Ralstonia*, *Bacillus*, *Virgibacillus* and *Clostridium_sensu_stricto_1*. In Group AC and Group AX, the dominant genera included *Corynebacterium*, *Leisingera*, *Romboutsia*, *Turicibacter* and *Nautella*, whereas *Staphylococcus*, *Bacillus*, *Virgibacillus* and *Geobacillus* were present at low abundance. Compared with Group AC, Group AX had significantly higher relative abundance of *Corynebacterium*, *Leisingera*, *Romboutsia*, *Turicibacter*, *Nautella* and *Bifidobacterium*, while the abundance of *Clostridium_sensu_stricto_1*, *Holdemanella*, *Dialister*, *Collinsella*, *Agathobacter* and *Blautia* decreased significantly. As shown in Figure 10D, 10 genera showed significant differences among the four groups, including *Corynebacterium*, *Leisingera*, *Turicibacter*, *Nautella*, *Clostridium_sensu_stricto_1*, *Romboutsia*, *Photobacterium*, *Ruegeria*, *Shimia* and *Rubritalea*. The relative abundance of *Corynebacterium* was significantly lower in the ammonia nitrogen stress groups (Group AC and Group AX) than in the normal groups (Group C and Group X), whereas the other nine genera were significantly enriched under ammonia nitrogen stress. Furthermore, the abundance of *Corynebacterium*, *Turicibacter*, *Nautella*, *Romboutsia*, *Shimia* and *Rubritalea* was significantly higher in Group AX than in Group AC, and the remaining four genera showed significantly lower abundance in Group AX. As shown in Figure 10E, samples from Group C and Group X clustered closely, indicating highly similar gut microbiota compositions in hybrid groupers of these two groups. After 24 h of ammonia nitrogen stress, the gut microbiota composition in Group AC and Group AX differed markedly from that in Group C and Group X, suggesting that ammonia nitrogen stress profoundly altered the intestinal microbiota of hybrid groupers. The variations of 13.37% and 73.30% in Group AC and Group AX were explained by PC2 and PC1, respectively.

## 4. Discussion

The results of the present study indicated that ammonia nitrogen toxicity to hybrid groupers is not limited to a single type of lesion but may manifest as multiple adverse outcomes including gut microbiota dysbiosis, intestinal tissue damage, disrupted serum physiological responses (antioxidant capacity, non-specific immunity and anti-stress capacity), and altered hepatic metabolic and immune signaling pathways. Collectively, these multi-dimensional datasets support the existence of an integrated toxic mechanism triggered by ammonia nitrogen. Microbiota data (Figure 10) verified that ammonia nitrogen stress reshaped the gut microbial community structure: the relative abundance of Actinobacteria decreased, while Firmicutes and Proteobacteria were markedly enriched. Meanwhile, beneficial *Bifidobacterium* declined, and the opportunistic pathogenic *Clostridium sensu stricto* underwent massive proliferation. Functional interpretation of microbial profiles indicated that elevated Proteobacteria is associated with aquatic stress-induced intestinal dysbiosis and low-grade chronic inflammation in fish [17,18,19]. Increased Proteobacteria abundance has been reported to exacerbate inflammatory infiltration in the intestinal epithelium, is known to disrupt tight junction architecture, can elevate intestinal permeability, and may facilitate translocation of endotoxins into the bloodstream [20,21]. By contrast, the elevation of Firmicutes under ammonia nitrogen stress represented merely a compensatory fluctuation following microbial structural disorder, without homeostatic protective effects, and failed to counteract Proteobacteria-mediated intestinal injury. Ammonia nitrogen stress aggravated intestinal dysbiosis and ultimately triggered multi-organ physiological dysfunction [19,22]. As illustrated in Figure 9E–H, ammonia-challenged fish exhibited time-dependent intestinal lesions, characterized by thinned intestinal walls, broken villi, depleted goblet cells and aggravated inflammatory infiltration. Toxic metabolites derived from disturbed microbiota are considered to disrupt epithelial tight junctions and potentially impair intestinal barrier integrity [21,23]. Impaired intestinal barrier may allow endotoxins to enter systemic circulation, which could stimulate excessive mitochondrial ROS release, subsequently disturbing whole-body redox homeostasis, leading to MDA accumulation and altering the balance of antioxidant enzymatic systems [21,24,25,26]. This observation is consistent with the changing profiles of antioxidant biomarkers detected in our study. In addition, continuous hepatic exposure to circulating toxins induces hepatocellular injury, massive leakage of AST, ALT and AKP, and suppresses lysozyme-governed non-specific immune defense [27,28,29]. Upon exposure to exogenous stressors, fish exhibit elevated cortisol levels, and the liver activates gluconeogenesis to sharply boost blood glucose, generating sufficient energy reserves to counteract stress-induced tissue damage [30]. Nevertheless, prolonged ammonia nitrogen stress renders endogenous de novo glucose synthesis insufficient to offset ammonia-mediated stress injuries. The organism consequently shifts to lactate as the primary substrate for gluconeogenesis, and excessive lactate consumption predisposes the fish to energy metabolic exhaustion [30,31]. Transcriptomic data further uncovered the molecular toxic mechanisms of ammonia nitrogen. The comparison between the control group and ammonia-stressed group (C vs. AC) displayed significant enrichment of pathways related to small molecule metabolism, alcohol metabolism, cytochrome P450 xenobiotic detoxification, and proteasome-mediated degradation [32]. Among these pathways, dysregulated steroid biosynthesis, aberrant mitochondrial protein metabolism, and disrupted complement and coagulation cascades are suggested to be potential molecular drivers of impaired ammonia detoxification capacity: disordered mitochondrial metabolism attenuates ROS scavenging efficiency and amplifies oxidative damage [33,34]; imbalanced steroid metabolism disturbs osmoregulation and stress hormone homeostasis in fish; and suppressed complement signaling impairs innate immune clearance capability. Collectively, these three cascades block hepatic ammonia detoxification and cause persistent toxin accumulation, thereby providing insights into the intrinsic molecular basis underlying ammonia-triggered metabolic collapse and immunosuppression at the transcriptional level, suggesting potential correlations between molecular pathway perturbations and macroscopic physiological lesions [35,36].

The present study suggests that dietary xylo-oligosaccharide (XOS) supplementation can effectively remodel gut microbial community composition, alleviate intestinal barrier damage, modulate serum physiological responses, and enrich hepatic transcriptomic pathways associated with immune and stress regulation, thereby potentially enhancing ammonia nitrogen tolerance in hybrid groupers to a certain extent. As shown in Figure 10, compared with the ammonia-challenged control group (Group AC), the XOS-supplemented ammonia-stressed group (Group AX) exhibited significantly reduced relative abundance of Proteobacteria, elevated proportion of Firmicutes, and enriched beneficial genera that maintain gut homeostasis including *Bifidobacterium*, *Turicibacter*, and *Romboutsia*, which robustly suppress the colonization of harmful taxa [8,37,38]. Numerous prior studies have documented that genera such as *Bifidobacterium*, *Turicibacter*, and *Romboutsia* facilitate gut short-chain fatty acid synthesis, optimize lipid metabolism, and sustain mucosal immune homeostasis [39,40,41]. These findings collectively suggest that XOS can efficiently modulate gut microbial structure and mitigate ammonia-induced disruption of gut microecological stability. As illustrated in Figure 9M–P, hybrid groupers fed XOS diets (Group AX) only presented mild intestinal lesions after 12 h of ammonia nitrogen exposure, without severe pathological alterations even at the 96 h stress time point. This protective phenotype is likely partially mediated by the XOS-enriched beneficial bacteria represented by *Bifidobacterium*. These commensal microbes ferment indigestible carbohydrates to produce acetate, propionate, and butyrate. As the primary energy substrate for intestinal epithelial cells, butyrate markedly accelerates epithelial proliferation, restores tight junction architecture, and alleviates ammonia-triggered intestinal apoptosis and inflammatory injury [42,43]. Attenuated intestinal barrier impairment is expected to restrict systemic translocation of endotoxins, thereby potentially reducing whole-body oxidative burden and improving organismal antioxidant capacity, non-specific immunity, and stress regulation (Figure 3, Figure 4 and Figure 5). In terms of antioxidant regulation, glutamine acts as a critical precursor for glutathione (GSH) synthesis. Endogenously abundant GSH cooperates with GSH-Px and CAT to eliminate excess reactive oxygen species (ROS), alleviating ammonia-stimulated lipid peroxidation and MDA accumulation [44,45]. The HIF-1 signaling pathway mediates glycolysis and gluconeogenesis to sustain cellular energy supply under stress conditions [46,47,48]. Dietary XOS supplementation likely up-regulates glutamine-synthesis-related metabolic pathways to boost systemic GSH contents, strengthening the GSH-Px-centered antioxidant defense system. Meanwhile, XOS co-activates the HIF-1 pathway to facilitate glycolytic energy production, persistently restraining ROS burst and massive MDA deposition during ammonia nitrogen stress. Regarding non-specific immunity, existing research has validated that ammonia exposure impairs immune barriers in fish spleen and head kidney, suppresses lysozyme activity to induce immunosuppression, and triggers massive secretion of pro-inflammatory cytokines that further provoke hepatic inflammatory lesions [49,50,51]. Fundamental immunological evidence demonstrates that Toll-like receptor and Jak-STAT signaling pathways coordinately govern the secretory balance of pro-inflammatory mediators such as TNF and IL-17, serving as core cascades maintaining systemic immune homeostasis [52,53,54]. From the perspective of stress modulation, ammonia nitrogen stress disrupts endocrine homeostasis and elicits abnormal elevation of serum cortisol. Excess cortisol secretion disturbs hepatic gluconeogenesis and destabilizes blood glucose balance [55,56,57,58], exacerbating stress-mediated physiological damage. Nevertheless, functional feed additives have been proven to stabilize serum cortisol concentrations and ameliorate stress-induced physiological disorders [54,59], which aligns well with our present results.

## 5. Conclusions

This study verified that ammonia nitrogen stress severely impairs intestinal histological morphology, triggers gut microbiota dysbiosis, and potentially disrupts the overall physiological homeostasis of hybrid groupers. Dietary xylo-oligosaccharide (XOS) supplementation could partially ameliorate multiple physiological traits, including systemic antioxidant capacity and anti-stress immune responses. This tendency is supported by elevated activities of serum functional enzymes and the enrichment of diverse functional cascades in the hepatic transcriptome. Transcriptomic annotation further revealed significant enrichment of pathways associated with immune-inflammatory modulation, oxygen homeostasis, and ammonia nitrogen metabolism, which may partially explain the alleviation of ammonia-triggered oxidative damage and inflammatory responses in XOS-fed hybrid groupers. Consistent with intestinal histology and gut microbiota data, XOS administration is associated with preserved intestinal barrier integrity and elevated abundance of commensal beneficial genera. Such reshaped gut microecological profiles contribute to improved intestinal health and partially attenuate tissue lesions caused by ammonia nitrogen exposure. In conclusion, XOS exhibits promising protective potential against ammonia nitrogen stress in hybrid groupers. Multi-dimensional evidence integrating gut microbiota, intestinal histopathology, serum physiological biomarkers and hepatic transcriptomic profiles collectively suggests that XOS alleviates the adverse impacts of ammonia stress via multi-layered regulatory networks. Nevertheless, the multi-omics data obtained in this study only demonstrate correlative relationships, and definitive causal links between XOS intervention and the modulation of ammonia metabolism or immune signaling pathways cannot be established. Further targeted functional assays are, therefore, required to validate these mechanistic associations.

## Figures and Tables

**Table 1:** Commercial feed nutritional composition.

Component	Content
Crude Protein	≥49.0%
Crude Lipid	≥9.0%
Crude Fiber	≤2.0%
Ash	≤16%
Moisture	≥2.5%
Dry Matter	≤10.0%
Total Phosphorus	1.5∼3.0%

**Table 2:** Primer sequences used for qPCR analysis.

Gene ID	Gene Name	Primer Sequence (5′–3′)
β-actin	β-actin	F:ATGAAACCACCTACAACAGT R:TAGACCCACCAATCCAGACG
TRINITY_DN3674_c0_g1	GST	F:CCAGGGAATTTCTCCTCCAAC R:GAGAACATTCAGCGTAAAGCAAAG
TRINITY_DN5303_c0_g1	UGT	F:TGTTCTTCTCCTCGTATTTCTTGTC R:CATTTCATTCAGTTACTCAGCCTTTC
TRINITY_DN406_c0_g1	HSP90	F:AGTCAATGCGACGGGAATAAG R:CTGCCAGCGTCCACAAGTAG
TRINITY_DN337_c0_g1	IL-1β	F:CCTTTCAGAGTGACGGCGAG R:GTGACCGAAACAGTCGTGGAG
TRINITY_DN2996_c0_g1	CXCL12	F:CGCATAATCACAGCCACAGAAC R:GACTTTGCCAGGTCGTAACTCAT
TRINITY_DN7254_c0_g3	MYD88	F:GTAACCAAGGACCAGGCAGTTAATG R:CAATCAGAAGCCAGCAGGATGTAG
TRINITY_DN3087_c0_g2	HIF1A	F:TTGACTGACCCACAAGAAAGAGG R:CCATTGATACAGAGCCGAAGAC
TRINITY_DN19260_c0_g2	IL6	F:GGCTTCTGAACCAGCTTGACC R:GAAACTACGGACGCAGGCAC

## Data Availability

The raw data of liver transcriptome (The BioProject accession number: PRJNA1442211) and 16S rRNA high-throughput sequencing (The BioProject accession number: PRJNA1441885) in this study have been uploaded to the NCBI Sequence Read Archive (SRA). The data are publicly available upon publication of this article.
